# Analysis for prevalence and physical linkages amongst integrons, ISE*cp*1, IS*CR*1, Tn*21* and Tn*7* encountered in *Escherichia coli* strains from hospitalized and non-hospitalized patients in Kenya during a 19-year period (1992–2011)

**DOI:** 10.1186/1471-2180-13-109

**Published:** 2013-05-17

**Authors:** John Kiiru, Patrick Butaye, Bruno M Goddeeris, Samuel Kariuki

**Affiliations:** 1 Centre for Microbiology Research, Kenya Medical Research Institute, PO Box 19464-00202, Nairobi, Kenya; 2 Department of Biosystems, Faculty of Bioscience Engineering, Katholieke Universiteit Leuven, Kasteelpark Arenberg 30, Heverlee, B-3001, Belgium; 3 Veterinary and Agrochemical Research Centre, Groeselenberg 99, Ukkel, B-1180, Belgium; 4 Department of Pathology, Bacteriology and Poultry Diseases, Faculty of Veterinary Medicine, University of Ghent, Salisburylaan 133, Merelbeke, 9820, Belgium; 5 Department of Virology, Parasitology and Immunology, Faculty of Veterinary Medicine, University of Ghent, Salisburylaan 133, Merelbeke, 9820, Belgium

## Abstract

**Background:**

We determined the prevalence and evidence for physical linkage amongst integrons, insertion sequences, Tn*21* and Tn*7* transposons in a collection of 1327 *E. coli* obtained over a 19-year period from patients in Kenya.

**Results:**

The prevalence of class 1 integrons was 35%, class 2 integrons were detected in 3 isolates but no isolate contained a class 3 integron. Integron lacking the 3’-CS or those linked to *sul*3 gene or IS*26* or those containing the IS*CR*1 were only detected in multidrug resistant (MDR) strains. The *dfrAs* were the most common cassettes and their prevalence was: - *dfrA*1(28%), *dfrA*12(20%), *dfA*17(9%), *dfrA*7(9%), and *dfrA*16(5%). The *aadA* were the second most abundant cassettes and their prevalence was: - *aadA*1(25%), *aadA*2(21%), and *aadA*5(14%). Other cassettes occurred in lower prevalence of below 5%. Prevalence of Tn*21*, ISE*cp*1, IS*CR*1 and IS*26* was 22%, 10%, 15%, and 7% respectively. Majority of Tn*21* containing integrons carried a complete set of transposition genes while class 2 integrons were borne on Tn*7* transposon. The *qnrA* genes were detected in 34(3%) isolates while 19(1%) carried *qnrB*. All *qnr* genes were in MDR strains carrying integrons containing the IS*CR*1. Close to 88% of *bla*_*TEM-*52_ were linked to IS*26* while ≥ 80% of *bla*_*CTX-M*s_ and *bla*_*CMYs*_ were linked to ISE*cp*1. Only a few studies have identified a *bla*_*CTX-M*-9_ containing an ISE*cp*1 element as reported in this study. Multiple genetic elements, especially those borne on *incIl*, *incFII*, and *incL/M* plasmids, and their associated resistance genes were transferrable *en bloc* to *E. coli* strain J*53* in mating experiments.

**Conclusions:**

This is the first detailed study on the prevalence of selected elements implicated in evolution of resistance determinants in a large collection of clinical *E. coli* in Africa. Proliferation of such strains carrying multiple resistance elements is likely to compromise the use of affordable and available treatment options for majority of poor patients in Africa. There is therefore a need to monitor the spread of these highly resistant strains in developing countries through proper infection control and appropriate use of antimicrobials.

## Background

Recent studies conducted in Kenya show that a significant proportion of *E. coli* strains from clinical specimens exhibit a strong multi-drug resistance (MDR) phenotype [1,2]. Fortunately, β-lactams, fluoroquinolones and aminoglycosides remain effective against a significant proportion of clinical *E. coli* strains in Kenya. However, recent studies have reported carriage of plasmid-borne *aac(6')-lb-cr* and *qnr* genes among β-lactamase producers [1,2]. The *qnr* genes confer resistance to quinolones*,* while *aac(6')-lb-cr* confers reduced susceptibility to fluoroquinolones and aminoglycosides. Therefore, carbapenems remain some of the few alternative antimicrobials that are effective against strains harboring a combination of multiple β-lactamase (*bla*) genes and genes conferring broad-spectrum resistance to fluoroquinolones and aminoglycosides. Carbapenems may however not be readily available or affordable for many patients in Sub-Saharan Africa [3].

In a recent study, we reported carriage of integrons, IS elements, Tn*21* and Tn*7* in a collection of 27 *E. coli* strains obtained from hospitalised patients [1]. These strains also harbored conjugatively transferrable plasmids conferring resistance to β-lactams, fluoroquinolones, aminoglycosides and co-trimoxazole among other antimicrobials suggesting that genes encoding resistance to these antimicrobials are physically linked to each other. Carriage of physically linked elements, each containing a set of resistance genes, may increases the chances of *en bloc* horizontal transfer of multiple resistance determinants to susceptible strains. Carriage of multiple resistance elements may in turn confer unique advantages to the host and enable them survive a strong antimicrobial selection pressure especially in hospital settings [4].

Studies to determine the prevalence of resistance elements in a large collection of strains from Sub-Saharan Africa are still lacking. Furthermore, little is known on whether the genetic elements encountered among *E. coli* strains in this region are physically linked to each other. In this study, we determined the prevalence of integrons, ISE*cp*1, IS*CR*1, IS*26* as well as transposons Tn*21* and Tn*7* in a collection of 1327 *E. coli* strains obtained from inpatient and outpatient populations seeking treatment in Kenyan hospitals during a 19-year period (1992–2011). We also determined genetic content of integrons and determined plasmid incompatibility groupings among strains exhibiting unique resistance phenotypes. Physical linkages among these elements and to *bla* genes were investigated using PCR methods. Similar analysis were done to determine if the *aac*(6')-*lb-cr* and *qnr* genes are physically linked to these elements.

## Results

### Antimicrobial susceptibility profiles

At least 25% of the 1327 isolates were resistant to expanded-spectrum β-lactams such as aztreonam (AZT), ceftriaxone (CRO), cefotaxime (CTX) and amoxicillin-clavulanic acid (AMC) combunation and to none-β-lactams such as streptomycin (S), nitrofurantoin (F), chloramphenicol (C), sulfamethoxazole (SUL), tetracyclines (TET) and trimethoprim (TRIM), Table 1. Resistance to a combination of two β-lactamase inhibitors, AMC and pipperacillin-tazobactam (TZP), was recorded in 22% of the isolates while 20% and 9% exhibited an ESBL- or an AmpC-like phenotype respectively, Table 2. A total of 106 strains were resistant to combinations of SUL, TRIM, ciprofloxacin (CIP), cefepime (FEP), gentamicin (CN), cefoxitin (FOX) and TZP. These isolates were therefore identified as strains with the highest potential to limit therapeutic option in clinical settings. Imipenem (IMI), cefepime FEP and CIP were effective against ≥ 90% of isolates. Strains from urine were more likely to exhibit an MDR phenotype compared to those from stool (p:0.0001, CI:27.2 to 84.8, OR:42) or blood (p:0.0001, CI:12.8 to 30.8, OR:19.9). Similarly, MDR phenotypes were more common among strains from hospitalized patients than those from non-hospitalized patients (p:0.0001, CI: 4.0 to 6.6, OR:5.1).

**Table 1:** Susceptibility profiles of isolates and their distribution in various specimen-types obtained from different categories of patients

			**Distribution [Number,%] of resistant strains in different specimen types**			**Distribution [Number,%] of resistant strains according to patient category**	
	**Number of resistant strains n = 1327**	**% of resistant strains**	**Stool n = 505**	**Urine n = 451**	**Blood n = 371**	**Inpatient n = 654**	**Outpatient n = 673**
**AMOX**	756	57	225 (30)	355 (57)	176 (23)	439 (58)	318 (42)
**AMP**	809	61	253 (31)	373 (46)	184 (23)	518 (64)	292 (36)
**AMC**	478	36	143 (30)	249 (52)	86 (18)	329 (69)	148 (31)
**AMS**	544	41	153 (28)	288 (53)	103 (19)	343 (63)	201 (37)
**TZP**	279	21	85 (30)	141 (51)	53 (19)	226 (81)	53 (19)
**AZT**	385	29	121 (31)	191 (50)	73 (19)	258 (67)	127 (33)
**CEF**	411	31	121 (29)	256 (62)	34 (8)	234 (57)	177 (43)
**CRO**	358	27	97 (27)	184 (51)	78 (22)	266 (74)	93 (26)
**CTX**	372	28	102 (27)	197 (53)	73 (19)	290 (78)	82 (22)
**CAZ**	279	21	83 (30)	142 (51)	54 (19)	201 (72)	78 (28)
**FEP**	119	9	31 (26)	76 (64)	12 (10)	99 (83)	20 (17)
**FOX**	106	8	19 (18)	79 (74)	8 (6)	87 (82)	19 (18)
**NA**	239	18	86 (36)	132 (55)	21 (9)	163 (68)	77 (32)
**CIP**	106	8	19 (18)	79 (75)	8 (8)	65 (61)	41 (39)
**STRP**	491	37	145 (30)	271 (55)	75 (15)	290 (59)	201 (41)
**K**	305	23	85 (28)	167 (55)	53 (17)	195 (64)	110 (36)
**CN**	239	18	71 (30)	131 (54)	37 (16)	170 (71)	69 (29)
**NEO**	212	16	71 (34)	120 (56)	21 (10)	174 (82)	38 (18)
**F**	385	29	89 (23)	254 (66)	42 (11)	277 (72)	108 (28)
**C**	478	36	167 (35)	233 (49)	78 (16)	320 (67)	158 (33)
**SUL**	637	48	189 (30)	356 (56)	92 (14)	440 (69)	197 (31)
**TET**	703	53	218 (31)	353 (50)	132 (19)	478 (68)	225 (32)
**TRIM**	557	42	167 (30)	290 (52)	100 (18)	379 (68)	178 (32)

The distribution of resistant strains in different specimen-types obtained from inpatients and outpatients. The percentages are calculated based on the total number of strains resistant to a given antimicrobial in different specimen types and category of patients. AMOX: amoxicillin, AMP: ampicillin, AMC: amoxicillin-clavulanic acid, AMS: ampicillin-sulbactam, TZP: piperacillin-tazobactam, AZT: Aztreonam, CEF: cefuroxime, CRO: ceftriaxone, CTX: cefotaxime, CAZ: ceftazidime, FEP: cefepime, FOX: cefoxitin, NA: nalidixic acid, CIP: ciprofloxacin, STRP: streptomycin, K: kanamycin, CN: gentamicin, NEO: neomycin, F: nitrofurantoin, C: chloramphenicol, SUL: sulfamethoxazole, TET: Tetracylines, TRIM: Trimethoprim.

**Table 2:** Distribution of isolates exhibiting combined resistance to selected antimicrobials

	**Total isolates exhibiting a given phenotype**	**Stool**	**Urine**	**Blood**	**Inpatient**	**Outpatient**
SUL, TRIM, CIP + CN + FEP + FOX + TZP and aminoglycosides ^a^	106	30 (28)	57 (54)	19 (18)	87 (82)	19 (18)
F + SUL + TRIM + TET + C^b^	451	121 (27)	233 (52)	97 (22)	322 (71)	129 (29)
AMC + AMS^c^	411	125 (30)	218 (53)	68 (17)	255 (62)	156 (38)
AMS + AMC + TZP^c^	291	87 (30)	172 (59)	32 (11)	194 (67)	97 (33)
ESBL strains	272	95 (35)	133 (49)	44 (16)	188 (69)	84 (31)
Isolates with an AmpC-like phenotype	122	38 (31)	72 (59)	12 (10)	93 (76)	29 (24)

Distribution of strains resistant to different combinations of antimicrobials among different specimen-types obtained from inpatient and outpatients. CIP: ciprofloxacin, CN: gentamicin, FEP: cefepime, FOX: cefoxitin, TET: tetracyclines, TZP: piperacillin-tazobactam, F: nitrofurantoin, SUL: sulfamethoxazole, TRIM: Trimethoprim, C: chloramphenicol, AMC: amoxicillin-clavulanic, AMS: ampicillin-sulbactam.

ESBL strains are susceptible to AMC and cephamycins but resistant to various combinations of cephalosporins while isolates with an AmpC-like phenotype are resistant to cephalosporins and cephamycins.

a: Isolates were resistant to at least one aminoglycoside.

b: These antimicrobials are relatively cheap and are readily available in developing countries.

c: Combinations of β-lactamase inhibitors that may be used to treat infections caused by strains that are resistant to β-lactams.

### Prevalence of integrons and integron cassettes

Class 1 integrons were detected in 35% of all isolates, 3 isolates carried class 2 integrons but none tested positive for class 3 integrons. The *dfrA* sub-types conferring resistance to TRIM and the *aadA*-type cassettes conferring resistance to aminoglycosides were the most common cassettes in class 1 and 2 integrons, Table 3. The prevalence of cassettes encoding resistance to trimethoprim was: - *dfrA*1 (28%), *dfrA*12 (20%), *dfA*17 (9%), *dfrA*7(9%), and *dfrA*16 (5%), while that of *aadA* cassettes conferring resistance to aminoglycosides was as follows: - *aadA*1 (25%), *aadA*2 (21%), and *aadA*5 (14%). Despite a relatively high prevalence of resistance to β-lactams, only *bla*_*OXA*-1_ was identified as an integron cassette. While *aadA* and *dfrA* types were detected in strains exhibiting resistance to between 2 and 8 classes of antimicrobials, *dfr*B, a*adA*5, *bla*_*OXA*-1_, *aac(*6’*)-lb-cr,* and *arr*2 were detected only in strains resistant to at least 6 different classes of antimicrobials. Majority (78%) of *dfrA*17 were detected in strains resistant to multiple generations of β-lactams.

**Table 3:** Diversity of cassette arrays detected among class 1 and class 2 integrons

				**Distribution [number, (%)] of cassette arrays of cassette arrays in different types of integrons**	
	**Resistance to selected antimicrobials in randomly selected strains carrying a given integron array**	**Classes of antimicrobials to which the host strain was resistant**^**a**^	**Prevalence among isolates with integrons (n = 464)**	**Integrons containing 3’-CS**	**Integrons lacking 3’-CS**
**Class 1 integrons arrays**					
*dfrA*1	**TRIM**, SUL, TET,	2 to 4	60 (13)	53 (88)	7 (12)
*dfrA*1/*aadA*1	**TRIM, STP**, AMP, C, CTX, CAZ, CIP, NA	5 to 8	51 (11)	42 (82)	9 (18)
*dfrA*17/*aadA*5	**TRIM, STP**, C, AMP, C, CTX, CAZ, CIP, NA, FOX, AMC	5 to 8	42 (9)	34 (81)	8 (19)
*dfrA*7	**TRIM**, SMX, TET	2 to 8	42 (9)	35 (83)	7 (17)
*aadA*1	**STP**, C, TET, SUL	2 to 6	23 (5)	19 (83)	4 (17)
*dfrA*12/*aadB*	**TRIM**, **STRP**, CN, K, TOB, AMP, C, CTX, AMC	4 to 8	23 (5)	19 (83)	4 (17)
*dfrA*16/*aadA*2	**TRIM, STP**, K, TOB, AMP, C, CTX, AMC	6 to 8	23 (5)	22 (96)	1 (4)
*aadA*2/*dfrA*12	**STP, TRIM**, TET, C, SUL, AMP, CTX, AMC,	3 to 6	28 (6)	26 (93)	2 (7)
*dfrA*12/*aadA*2	**TRIM, STP**, TET, C, SUL	3 to 8	23 (5)	22 (96)	1 (4)
*aadA*5	**STP**, AMP, SUL, TET	7 to 8	23 (5)	22 (96)	1(4)
*blaoxa*-1/*aadA*1	**STP**, **AMP**, C, TET, CTX, CAZ, CIP, NA, FOX, AMC	8	23 (5)	22 (94)	1 (4)
*blaoxa*-1/*aadA*2	**STP**, **AMP**, C, TET, CTX, CAZ, CIP, NA, FOX, AMC	7 to 8	9 (2)	8 (88)	1 (12)
*dfrA*12/*orfF*/*aadA*2	**TRIM, STP**, C, TET, CTX, NA, AMC	6 to 8	9 (2)	8 (88)	1 (12)
*aac*(6')*Ib*/*catB*1/*dfrA*1	CN**, TOB, C, TRIM**, **K**, AMP, C, TET, CTX, CAZ, CIP, NA,	5 to 8	9 (2)	7 (78)	2 (22)
*aadA*1/*dfrA*1	**STP, TRIM**, AMP, C, TET, CTX, NA, AMC	3 to 8	9 (2)	9 (100)	0
*aac*(6')*Ib*/*bla*_*oxa*-1_/*catB*3/*arr*2	CN,**TOB**, **K, C, RIF**, **AMP**, C, TET, CTX, CAZ, CIP, NA, FOX, AMC	8	9 (2)	2 (22)	7 (78)
*aadA*2/*orfF*/*dfrA*12	**STP**, AMP, **TRIM**, SUL, TET	7 to 8	5 (1)	4 (80)	1 (20)
*cmlA*1	**C,**, TET, CTX, NA, AMP	3 to 8	3 (<1)	3 (100)	0
*orf*5/dfrB/orfA	**TRIM,** CN,TOB, C, AMP, C, TET, CTX, CAZ, CIP, NA, AMC	6	3 (<1)	0	3 (100)
*dfrA*12/*aadA*1/*bla*_*o*xa1_	**TRIM, STP**, CN,TOB, **AMP**, C, TET, CTX, CAZ, CIP, NA,	8	5 (1)	0	5 (100)
*aac*(6')-*lb-cr*	CN, **K, TOB**, C, AMP, C, TET, CTX, CAZ, **CIP**, NA, AMC	8	42 (9)	15 (36)	27 (64)
**Class 2 Integron arrays**					
*drfA*1/*sat*2/*aadA*1	**TRIM**, **STRP**, CN, K, TOB, AMP, C, CTX, AMC	6 to 8	3 (<1)	NA	NA

The integron cassette arrays are indicated in the order they appear within class 1 and 2 integron variable cassette region (in the 5’-3’ orientation).

The resistance phenotype associated with a given array is indicated in bold.

a: Different antimicrobials tested in this study were conveniently grouped into 8 groups:- β-lactams and β-lactamase inhibitors, aminoglycosides, (fluoro)quinolones, nitrofurantoin, chloramphenicol, sulphonamides, trimethoprim, and tetracyclines.

b: These integrons carried a *sul*3 gene at the 3’-end or lacked this gene or 3’-CS comprising the *qacEΔ*1-*sul1* genes.

The *cmlA*1 and *aadA*1/*dfrA*1 cassette arrays were only detected in integrons containing a 3’-CS. In contrast, at least 64% of *aac*(6')-*lb-cr, dfrA*12/*aadA*1/*bla*_*o*xa1,_*orf*5/*dfrB*/*orfA*, and *aac*(6')*Ib*/*bla*_*oxa*-1_/*catB*3/*arr*2 cassette arrays were detected in integrons lacking typical 3’-conserved sequences (3’-CS) that contains *qacEΔ*1 (a truncated gene encoding resistance to quaternary ammonium compounds, and *sul*1 encoding resistance to sulfonamides). All the three class 2 integrons contained an identical cassette array comprising *dfrA*1-*sat*2-*aadA*1.

### Prevalence of Tn*21*, Tn*7* and IS elements

The prevalence of Tn*21* was 22% while Tn*7* was detected in 3 isolates that also carried class 2 integrons. Prevalence of ISE*cp*1, IS*CR*1 and IS*26* was 10%, 15%, and 7% respectively. A high proportion (≥ 60%) of isolates containing the IS elements and integrons were MDR (resistant to at least 3 different classes of antimicrobials), Table 4. Isolates carrying multiple elements were more likely to exhibit an MDR phenotype than those lacking such elements (p:0.0001, CI:549.5 to 2419.6, OR:1153) and isolates from urine were more likely to harbor multiple elements compared to those from blood (p:0.0001, CI:3.1 to 5.5, OR:4.1) or those from stool (p:0.0008, CI:1.2 to 2.0, OR:1.6). Although integrons, IS elements and Tn*21* were detected in isolates from all specimen-types, a high proportion (69%) of these elements were detected among strains from urine of hospitalized patients.

**Table 4:** **Carriage of resistance genetic elements among 1327*E. coli*exhibiting resistance to different classes of antimicrobials**

			**Classes of antimicrobials to which host strains were resistant**^**a**^			
**Combinations of genetic elements**	**Isolates positive for genetic elements**	**% among 1327 isolates**	**0**	**1 ≤ 2**	**3 ≤ 5**	**6-8**
Integrons	464	35	0	37 (8)	65 (14)	362 (78)
IS*CR*1	199	15	0	0	18 (9)	181 (91)
ISE*cp*1	128	10	0	0	35 (27)	93 (73)
IS*26*	86	7	0	0	12 (14)	74 (86)
Tn*21*	289	22	0	18 (6)	33 (11)	238 (83)
Tn*7*	3	<1	0	0	1 (25)	2 (75)
**Combination of genetic elements in same isolate**						
Integron + IS*CR*1 + Tn*21*	38	3	0	0	2 (5)	36 (95)
Integron + IS*CR*1 + IS*26*	28	2	0	0	2 (7)	26 (93)
Integron + IS*CR*1 + ISE*cp*1 + Tn*21*	16	1	0	0	0	16 (100)
No genetic element detected	332	35	307 (93)	25 (6)	0	0

Carriage of genetic elements or combination of elements among strains exhibiting resistance to different antimicrobials tested in this study. The antimicrobials were grouped into 8 convenient groups:- β-lactams and β-lactamase inhibitors, aminoglycosides, (fluoro)quinolones, nitrofurantoin, chloramphenicol, sulphonamides, trimethoprim, and tetracyclines.

### Physical linkage amongst genetic elements

**Figure 1:**
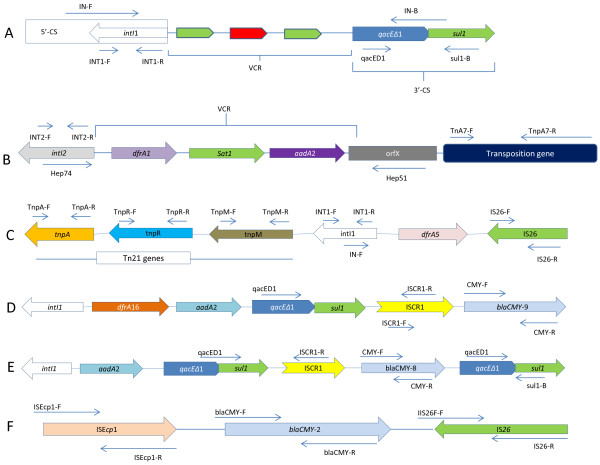
**Schematic diagram showing some of the strategies for screening for various genetic elements and for interrogation between these elements and resistance genes.** The targets of each primer and the direction of PCR amplification is shown using arrows. PCRs were done both in the 5’ and in the 3’ orientation for each pair of genes tested. **A**: The strategy used for detection and characterization of class 1 integrons. **B**: The strategy used for detection and characterization of class 2 integrons and their physical linkage to Tn*7*. **C**: An example of the strategy used for analysis of physical linkages between class 1 integrons and Tn*21* and to IS*26*. The primer positions for screening of Tn*21* transposition genes. **D** and **E**: An example of the strategy used for analysis for physical linkages between integrons, IS*CR*1 and *bla* genes. **F**: An example of the strategy used for analysis for physical linkages between integrons, ISE*cp*1, IS*26* and *bla* genes. These illustrations are based on PCR mapping data and not sequencing. Therefore, the sizes of each gene and the distances between any two genes are not drawn to scale.

**Figure 2:**
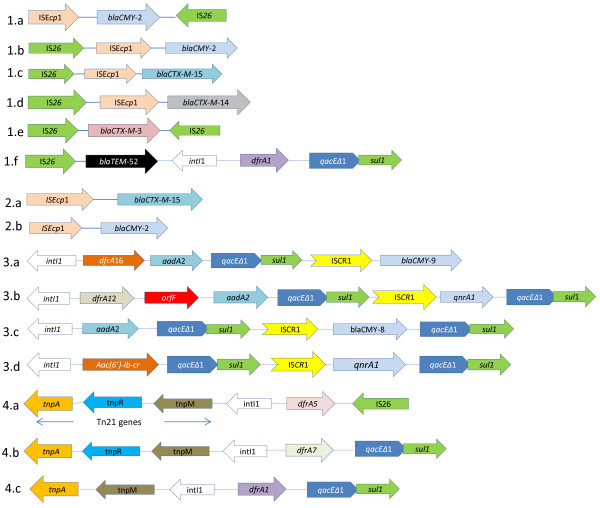
**Schematic diagram illustrating examples of physical linkages amongst genetic elements and selected genes. 1a-1f**: An example of physical linkages between *bla* genes and multiple genetic elements such as integrons, ISE*cp*1, and IS*26*. **2a-2b**: An example of physical linkages between *bla* genes and ISE*cp*1. **3a-3d**: An example of physical linkages between integrons and other genetic elements (such as the IS*CR*1 element) that are in turn linked to *bla* genes and (fluoro)quinolone resistant genes. **4a-4c**: An example of physical linkages between Tn*21* and integrons that are in turn be linked to IS elements. These illustrations are based on PCR mapping data and not sequencing. Therefore, the sizes of each gene and the distances between any two genes are not drawn to scale.

Figure 1 illustrates the strategy used for interrogation for physical linkages amongst genetic elements while Figure 2 illustrates some of the genetic associations identified in this study. Majority (69%) of integrons containing 3’-CS were physically linked to the Tn*21* transposon while 75% of those containing a *sul*3 gene at the 3’-terminal were linked to IS*26*. This element was also linked to 80% of integrons lacking the 3’-CS, Table 5. Forty (40) isolates contained class 1 integrons linked to a single IS*26* upstream the 5’-CS while in 12 isolates the integrons was flanked by two IS*26* elements. All ISCR1 were detected only in MDR strains and were flanked by a pair of class 1 integron 3’-CS. Close to 94% of Tn*21* that were linked to an integron contained a complete set of transposition genes (*tnpA, tnpR* and *tnpM*) while 89% of Tn*21 *with an incomplete set of these genes did not contain an integron, Table 6. All the three class 2 integrons were physically linked to Tn*7*.

**Table 5:** Physical linkages between integrons and other genetic elements

		**Integrons (number,%) physically linked to different elements**				
**Type of integrons**	**Total detected**	**Tn*7***	**Tn*21***	**IS*CR*1**	**ISE*cp*1**	**IS*26***
Class 1 integrons with 3‘-CS	375	3 (1)	257 (69)	199 (53)	19 (5)	4 (1)
Class 1 integron with *sul*3	64	0	12 (19)	0	12 (19)	48 (75)
Class 1 integrons lacking 3’-CS or *Sul*3	25	0	5 (20)	0	10 (40)	20 (80)
Class 2 integron	3	3 (100)	1 (33)	1 (33)	1 (33)	0

Carriage of Tn*21*, Tn*7* and IS elements among strains carrying class 1 integrons. Carriage of other genetic elements among strains carrying class 2 integrons is also shown.

**Table 6:** **Carriage of transposition genes among Tn*21*transposons**

		**Number (%) of Tn*21*transposition gene combination**		
**Category of Tn*21***	**Number of Tn*21*detected**	***tnpA*** **+** ***tnpM*only**	***tnpR*** **+** ***tnpM*only**	***tnpM*** **+** ***tnpA*** **+** ***tnpR***
Tn*21* linked to integrons	156	0	9 (6)	147 (94)
Tn*21* not linked to integrons	133	56 (42)	63 (47)	14 (11)

PCR methods were used for screening for three genes that are crucial for transposition of Tn*21*. The *tnpA* encodes a Tn*21*-like transposase, the *tnpM* encodes a putative transposition regulator. Integrons are incorporated into the Tn*21* framework adjacent to the *tnpM* gene. The *tnpR* encodes a resolvase.

### Physical linkages between resistance genes and genetic elements

Figure 2 illustrates selected examples of physical linkages between *bla* genes and different genetic elements. Over 40% of isolates carrying *bla*_*TEM-*52_, *bla*_*SHV*-5_ or *bla*_*CTX-M*-14_ were physically linked to the IS*26,* Table 7. The ISE*cp*1 was the most common IS element associated with *bla*_*CTX-M*-14,_*bla*_*CTX-M*__−15_ and *bla*_*CMY-*2._ One isolate contained a *bla*_CTX*-M*-9_ linked to this element. In all cases, the ISE*cp*1 was detected upstream the *bla* gene, Figure 2.

**Table 7:** **Analysis for physical association between*bla*genes and various genetic elements**

		**Number (%) of β-lactamase physically linked to various genetic elements**			
**β-lactamase genes**	**Number of isolates tested**	**IS*26***	**ISE*cp*1**	**IS*CR*1**	**Integrons**
*bla*_*SHV-1*_	60	23 (38)	12 (20)	10 (17)	9 (15)
*bla*_*OXA*-1_	43	12 (28)	21 (49)	32 (74)	36 (84)
*bla*_*OXA*-2_	17	0	2 (12)	5 (29)	3 (18)
*bla*_*SHV*-5_	18	10 (55)	5 (28)	3 (17)	1 (6)
*bla*_*SHV*-12_	19	7 (37)	4 (21)	3 (16)	2 (11)
*bla*_*CTX-M*-1_	9	1 (11)	0	2 (22)	1 (11)
*bla*_*CTX-M*-3_	15	6 (40)	0	0	0
*bla*_*CTX-M*-8_	6	2 (33)	1 (17)	0	0
*bla*_*CTX-M*-9_	3	0	1 (33)	3 (100)	0
*bla*_*CTX-M*-14_	25	10 (40)	3 (12)	5 (20)	3 (12)
*bla*_*CTX-M*-15_	32	4 (13)	30 (94)	0	0
*bla*_*TEM*-103_	18	2 (11)	0	1 (6)	1 (6)
*bla*_*TEM*-109_	9	0	0	0	0
*bla*_*TEM*-50_	10	2 (20)	1 (10)	6 (60)	3 (30)
*bla*_TEM-52_	37	29 (78)	1 (3)	3 (8)	2 (5)
*bla*_*TEM*-78_	9	2 (22)	0	3 (33)	2 (22)
*bla*_*TEM*-125_	36	3 (8)	0	3 (8)	2 (6)
*bla*_*TEM*-152_	14	1 (7)	0	4 (29)	2 (14)
*bla*_*TEM*-158_	10	1 (10)	0	0	0
*bla*_*CMY*-2_	48	12 (25)	42 (88)	12 (25)	3 (6)

Analysis for physical linkages between *bla* genes and various genetic elements. The *bla* content of the isolates analyzed had been determined in a past study [3].

Thirty seven (88%) of the 42 *aac(*6’*)-lb-cr* were borne on integrons containing the IS*CR*1 while 55% were borne on integrons linked to the IS*26*. Twenty four (71%) of the 34 isolates carrying a *qnrA* gene were resistant to nalidixic acid but not to ciprofloxacin while the other 10 isolates carrying this gene and 19 carrying the *qnrB* subtype were resistant to both antimicrobials, Table 8. None of the isolates tested positive for *qnrS*. Majority (87%) of *qnr* genes were physically linked to either integron-associated IS*CR*1 or the IS*26*. All Isolates carrying *aac(*6’*)-lb-cr* or the *qnr* genes contained multiple genetic elements and were all MDR.

**Table 8:** **Carriage of*aac(6')-lb-cr*and*qnr*genes among strains containing genetic elements and*bla*genes**

		**Number (%) of strains carrying each gene and number (%) of strains containing genes linked to genetic elements**								**Occurrence in strains carrying*bla*genes**^**a**^		
	**Total**	**Strains containing*intI1***	**Linked to*intI1***	**Strains containing IS*26***	**Linked to IS*26***	**Strains containing IS*CR*1**	**Linked to IS*CR*1**	**Strains containing ISE*cp*1**	**Linked to ISE*cp*1**	**β-lactamase negative strains**	**Strains containing TEM-1 or SHV-1 only**	**Strains containing broad-spectrum*bla*genes**
***aac(6’)-lb-cr***	42	42 (100)	42 (100)	6 (14)	4 (9)	12 (29)	6 (14)	11 (26)	4 (10)	0	4 (9)	38 (91)
***qnrA***	34	27 (79)	26 (75)	11 (32)	4 (12)	28 (82)	23 (68)	8 (24)	1 (3)	0	2 (6)	32 (94)
***qnrB***	19	19 (100)	11 (58)	10 (53)	2 (11)	13 (64)	4 (21)	12 (63)	1 (5)	0	1 (5)	18 (95)

Table shows the number of isolates carrying the three (fluoro)quinolone resistance genes and the proportion of such strains in which these genes were physically linked to various genetic elements and to *bla* genes.

a: Distribution of the *aac(*6’)*-lb-cr* and *qnr* genes among strains fully susceptible to β-lactams, among those resistant to TEM-1 or SHV-1 with a narrow substrate-range and among those carrying genes encoding broad-spectrum β-lactamases such as *bla*_*SHV*-5_, *bla*_*SHV*-12_, *bla*_*CMY*_ and *bla*_*CTX-Ms*_.

### Conjugative plasmids mediate *en bloc* transfer of multiple elements and resistance genes

Multiple resistance genes and genetic elements associated with them were transferred *en bloc* to *E. coli J53* in mating experiments, Table 9*.* Majority of such transferred were mediated by plasmids containing *I1*, *L/M*, *XI*, *HI*2 and the F-type replicons. These experiments further revealed that genes conferring resistance to tetracylines and chloramphenicol were also harbored in the same plasmids encoding resistance to β-lactams, (fluoro)quinolones and aminoglycosides. However, various gene combinations that had been determined to be physically linked using PCR could not be transferred in conjugation experiments using media containing different combinations of antimicrobials.

**Table 9:** **Horizontal transfer of genetic elements and associated resistance genes from clinical strains (donors) to*E. coli J53*(recipient)**

**Resistance profiles among donor and transconjugants**			
**Resistance to selected antimicrobials among donors**	**Physically linked genetic elements or resistance genes detected in donors and recipients**	**Other genes whose linkages were not determined**	**Plasmid replicons detected**
**AMP, CTX, CAZ, FOX**, NA, CIP, **TET**, C, **AMC**, K, CN, **SUL**	**ISE*cp*1/*bla***_***CMY*-2**_**/IS*26***	*aadA*1, *bla*_*SHV*-12_	P, **I1**
**AMP, CTX, CAZ, FOX, NA,** CIP**, TET,** C, **AMC,** K, CN, **SUL**	**IS*26*/ISE*cp*1/b*la***_***CMY*-2**_**,*qnrA*1**	*Tn21, dfrA*5, *sul*1	**L/M**
**AMP, CTX, CAZ,** NA, **TET, C**, AMC, **K, CN**, **SUL, TRIM**	**IS*26*/ISE*cp*1/*bla***_***CTX-M*-15**_	***Tn21, dfrA*1,*aac(6’)lb***	**FII**, F, A/C
**AMP, CTX,** CAZ**,** NA, TET, C, AMC, **K**, CN, **SUL,** TRIM	IS*26*/ISE*cp*1/*bla*_*CTX-M*-14_	***Tn21, aadA*5,*sul*1**, **b*laTEM***-1	A/C, **K**, B/O
**AMP, CTX, CAZ,** NA, TET, C, AMC, K**,** CN, **SUL,** TRIM	**IS*26*/*bla***_***CTX-M*-3**_**/IS*26***	*aac(6’)lb*, *qnrB*	**FII**, F
**AMP, CTX,** CAZ**,** NA, **TET,** C, AMC, K, CN, **SUL, TRIM**	**IS*26*/*bla***_***TEM*-52**_**/*intI*1/*dfrA*1/*qacE***Δ**1/sul1**	*bla*_*TEM*-1_	**I1**, FIB
**AMP, CTX,** CAZ**,** NA, CIP, TET**,** C, **AMC**, **K,** CN, **SUL, TRIM**	*ISEcp1/bla*_*CTX-M-*15_	***dfrA*12,*aadA*1,*bla***_***OXA*-1**_***bla***_***TEM*-1**_**,*sul*3**	**XI**
**AMP, CTX, CAZ, FOX**, **NA**, **CIP**, TET, **C**, **AMC**, **K, CN, SUL**	**ISE*cp*1/*bla***_***CMY*-2**_**/*intI*1/*aac(6')-lb-cr/*IS*CR*1/*qnrA*1**	*aac(6’)lb*, *catB*3, *dfrA*1	**L/M**, K
**AMP, CTX**, CAZ, NA, CIP, TET, C, AMC, K, CN, **SUL**, TRIM	i*ntI*1/*dfrA*16/*aadA*2/*qacE*Δ1/*sul*1/IS*CR*1/*bla*_*CTX-M*-9_	***bla***_***TEM-1***_*,****bla***_***SHV*-5**_	**L/M**
AMP, CTX, CAZ, NA, CIP, **TET**, C, AMC, K, CN, **SUL**, TRIM	intI1/*dfrA*12/*orf*F/*aadA*2/qacEΔ1/*sul*1/IS*CR*1/*qnrA*/*qacE*Δ1/*sul*1	*blaCTX-M*-15, *bla*_*TEM*-1_, *bla*_*OXA*-1_	I1, **FIB**
**AMP, CTX, CAZ, FOX**, **NA**, CIP, **TET**, **C**, **AMC**, **K, CN, SUL**	***intI*1/*aadA*2/q*acE***Δ**1/*sul*1/IS*CR*1/*bla***_***CMY*-2**_**/*qacE***Δ**1/*sul*1/IS*CR*1/**	*qnrA*1,	**I1**, K, B/O
**AMP, CTX,** CAZ, **NA, CIP**, TET, C, AMC, K, CN**,** TRIM **SUL**	**intI1/*aac(6')-lb-cr*/*qacE***Δ**1/*sul*1/*qnrA*1/*qacE***Δ**1/*sul*1**	***bla***_***TEM*-1**_**,*bla***_***SHV*-5**_	FIA, **FIB**
AMP, CTX, NA, CIP, **TET,** C, AMC, K**,** CN, **SUL, TRIM**	**Tn*21*/*intI*1/*dfrA*5/IS*26***	*bla*_*TEM*-125_	FIB, F, **HI2**
**AMP, CTX,** NA, CIP, **TET,** C, AMC, K, CN, **SUL**, **TRIM**	**Tn*21*/*intI*1/*dfrA*7/*qacE***Δ**1/*sul*1**	***bla***_***CTX-M*-8**_**,**	**I1**, F
**AMP, CTX,** CAZ, NA, CIP, **TET,** C, **AMC**, K, CN, **SUL**, TRIM	**Tn*21*/*intI*1/*dfrA1*/*qacE***Δ**1/*sul*1**	*bla*_*TEM-15*_, ***bla***_***TEM*-1**_, ***bla***_***OXA*-1**_, *aac(*6'*)-lb-cr*	FIB, **HI2**

Table shows carriage of genetic elements and selected genes conferring resistance to important classes of antimicrobials. The resistance phenotype and the genetic elements or genes transferred to the transconjugants are indicated in bold.

## Discussion

The current study shows that a significant proportion of clinical *E. coli* strains in Kenyan are resistant to important classes of antimicrobials such as β-lactams, fluoroquinolones and aminoglycosides. These results are in agreement with those published before [1,3,5]. These MDR strains were however susceptible to carbapenems. It is easy (although illegal) to purchase antimicrobials in Kenya without prescriptions or with prescriptions not backed by laboratory investigations [6]. We hypothesize that such practices may directly or indirectly lead to emergence of highly resistant strains.

A high prevalence of MDR strains from urine and all specimens from hospitalized patients may reflects a corresponding heavy use of antimicrobials among this category of patients as reported in past studies [7,8]. Majority of resistances encountered in hospital isolates were also encountered in community settings probably because patients are often discharged from hospitals as soon as their conditions improve, even before they complete their treatment regiments (our unpublished observations). It is therefore possible that hospital strains find their way into community settings and *vis versa.* However, we do not rule out the possibility that some MDR phenotypes may arise in community settings.

The high prevalence of class 1 integrons may partially be due to their association with the Tn*21* that contain a complete set of transposition genes. Past studies show that *dfrA*7 and *dfrA*1 cassettes associated with Tn*21*-borne integrons are the most prevalent *dfrA*-subtypes in Central, North and Western Africa [9-12]. In this study however, the prevalence of *dfrA*7 was much lower than that of *dfrA*1, *dfrA*12 and *dfA*17 in that order. The class 2 integron *dfrA*1/*sat*2/*aadA*1 array reported in this study is globally distributed [13]. Our results may therefore reflect regional differences or similarities in distribution of integron cassette arrays. Such differences may arise from unique antimicrobial-use patterns in different countries. This study also demonstrates an apparent correlation between carriage of *dfrA*17 and resistance to multiple β-lactams as has been reported in Tunisia [12,14] and from Northern Kenya among isolates from dog, cat and human specimens [5]. The reasons behind these correlations are yet to be elucidated. Carriage of different *dfrA* sub-types in our isolates and carriage of multiple integron-associated *sul* genes (*sul*1 and *sul*3) in the same isolate possibly correlates to heavy usage of sulfonamides and trimethoprim in Kenya for treatment of different infections and as prophylaxis against opportunistic infections among people with HIV/AIDS [15-17].

Some integrons, especially those lacking the 3’-CS and those containing a *sul*3 at the 3’-end, were linked to the IS*26* possibly because this element mediates deletion of 3’-CS in class 1 integrons 3’- terminal [18,19]. Similar results have been published in Australia, Spain and Nigeria [11,12,18,19]. Our data further suggest that strains carrying IS*26*-associated integrons are highly MDR probably because the IS*26* is also linked to other non-integron genes such as β-lactamases.

Most β-lactamases, particularly those encoding CTX-M-14 and −15 and CMY-2, were physically linked to ISE*cp*1. Similar reports have been published in Tunisian [20,21] but no ISE*cp*1 was detected upstream the *bla*_*-CTX-M*-1_ among our isolates as reported in a related study from the same country [22]. In one isolate, this element was found upstream the *bla*_*CTX-M*-9._ Reports of ISE*cp*1-*bla*_*CTX-M*-9_ linkages are rare but such linkages have been reported in *Klebsiella pneumoniae* isolates in Taiwan [23]. Majority of *bla*_*TEM*_ genes, *bla*_*TEM*-52_ in particular, were physically linked to the IS*26* as reported in Belgium and Germany [24,25]. Taken together, these results suggest that most *bla* genes in our isolates are in similar genetic environments as those reported globally but the genetic environment of *bla*_*CTX-M*-9_ and *bla*_*CTX-M*-1_ in our isolates appears to be different from those reported globally.

Our results further demonstrated that most *bla* genes are distantly linked to elements that are in turn linked to other resistance genes such as *aac*(6’)-*lb-cr* and *qnr*. Similar reports have been published in Tunisia [20,21] and in Nigeria [11]. ISE*cp*1, IS*26* and IS*CR*1 are known to mediate transposition and/or expression of multiple resistance genes in their close proximity [26-31]. Carriage of such multiple elements, each carrying a set of resistance genes may be responsible for the observed co-resistance to multiple antimicrobials among our isolates.

Conjugation experiments confirmed that multiple elements were borne on narrow host-range plasmids such as *IncFII*, *IncH1*2 or on broad host-range plasmids such as *IncL/M.* The type of conjugative plasmids in our isolates (especially those carrying plasmids containing *incF*-type, *incHI*2 and incI1 *incL/M* replicons) were shown to confer resistances similar to those in strains from Tunisia, [32] and from two other studies conducted in Kenya [1,5]. We hypothesis that plasmids of different incompatibility groups have acquired similar or identical sets of resistance genes and this acquisition is mediated by genetic elements such as those investigated in this study. Therefore, there is a possibility that such elements act as genetic shuttles between plasmids of different incompatibility grouping. The similarities and differences in genetic environments of *bla*, *aac (6’)-lb-cr* and *qnr* genes reported in this study may reflect a difference in transposition activities of such elements. We further hypothesize that differences in antibiotic use patterns in different regions influence the transposition activity of such elements.

## Conclusions

This study reports carriage of multiple genetic elements in MDR *E. coli* strains and their association with selected resistance genes. Strains carrying such elements are likely to be well adapted to survive deleterious effects of combined antimicrobial therapy. Furthermore, such MDR strains have a potential to increase morbidity and mortality among patients. It is therefore important to launch surveillance programs and to put up measures to curtail the spread of these highly resistant strains. There is also a need to compare the genomes of strains encountered in Africa with those from other parts of the world.

## Methods

### Isolates

The 1327 non-duplicate isolates were obtained sequentially from 13 healthcare facilities in Kenya between 1992 and 2011 (19-year period) from 654 hospitalized and 673 non-hospitalized patients. These isolates comprised of 451 strains from patients with urethral tract infections (UTI) and those with urinary catheters while 371 were from blood of patients with septicemia. Another 505 strains were from fecal specimens of patients with loose stool, watery and bloody diarrhea. Only one isolate per specimen per patient was included for further analysis. Among the isolates investigated in this study, 912 had been analyzed for *bla* genes in a a past study [3] while 27 had been analyzed for selected genetic elements [1]. Ethical clearance to carry out this study was obtained from the KEMRI/National Ethics Committee (approval number SSC No. 1177).

### Antimicrobial susceptibility profiles

Susceptibility profiles for all isolates were determined using antibiotic discs (Cypress diagnostics, Langdorp, Belgium) on Mueller Hinton agar (Oxoid, Louis, Mo. USA) using the Laboratory Standards Institute guidelines (CLSI) [33].

### Detection of genetic elements

Figure 1 illustrates the strategy used for detection and characterization of integrons and transposons. Detection of class 1, 2 and 3 and determination of carriage of 3’-conserved sequences (3’-CS) in class 1 integrons was done as described before [34,35]. Class 1 integron variable cassette region (VCR), the region in which the resistance gene cassettes are integrated, was amplified as previously described by Dalsgaard *et al.*[35] while that of class 2 integrons was amplified as described by White *et al.*[36]. The VCRs of integrons lacking the typical 3’-CS was determined using a PCR walking strategy published before [37]. Identification of integron cassette identity was done using a combination of restriction fragment length polymorphism (RFLP), sequencing and published bioinformatics tools [38,39]. Detection of the IS*Ecp*1, IS*CR*1, Tn*21* and Tn*7* elements was done as described in published studies [34,35]. Analysis for Tn*21* transposition genes:- *tnpA, tnpR* and *tnpM* genes was done as previously described by Pearson *et al.*[40]. The primers used in this study are presented in Table 10.

**Table 10:** Primers for screening for genetic elements and resistance genes and for analysis for physical linkages among such elements and selected resistance genes

**Target Gene/region**	**Primer name**	**5'-3' sequence**	**Annealing Temperature**	**Expected product size (bp)**	**Gene accession Number**
**Integrons**					
*intI*1	INT-1 F	GTTCGGTCAAGGTTCTG	50	923	U12338
	INT-1R	GCCAACTTTCAGCACATG			
*intI*2	INT-2 F	ATGTCTAACAGTCCATTTT	50	450	AJ001816.1
	INT-2R	AAATCTTTAACCCGCAAAC			
*intI*3	INT3-F	GCAGGGTGTGGACGAATACG	57	760	AY219651
	INT3-R	ACAGACCGAGAAGGCTTATG			
3'-CS	qacED1	ATCGCAATAGTTGGCGAAGT	56	800	X15370
	sul1-B	GCAAGGCGGAAACCCGCGCC			X12869
integron class 1 VCR	In-F	GGCATACAAGCAGCAAGC	52	Variable	U12338
	In-B	AAGCAGACTTGACCTGAT			
integron 2 VCR	hep74	CGGGATCCCGGACGGCATGCACGATTTGTA	55	Variable	EU780012
	hep51	GATGCCATCGCAAGTACGAG			AJ002782
**IS elements**					
ISE*cp*1	ISEcp1-F	GTT GCT CTG TGG ATA ACT TG	55	180	AJ242809
	ISEcp1-R	CCT AAA TTC CAC GTG TGT			
IS*CR*1	ISCR1-F	CGC CCA CTC AAA CAA ACG	55	469	L06418
	ISCR1-R	GAG GCT TTG GTG TAA CCG			
IS*26*	IS26-F	GCGGTAAATCGTGGAGTGAT	55	704	NC 007941.1
	IS26-R	ATTCGGCAAGTTTTTGCTGT			
**Tn21 and Tn7**					
*tnpM* of Tn*21*	TnpM-F	TCAACCTGACGGCGGCGA	55	348	AF071413
	TnpM-R	GGAGGTGGTAGCCGAGG			
*tnpR* of Tn*21*	TnpR-F	GTC AGC AGC TTC GAC CAG AA	62	500	NC 002134.1
	TnpR-R	GAG GTA CTG GTA GAG GGT TT			
*tnpA* of Tn*21*	TnpA21-F	TGC GCT CCG GCG ACA TCT GG	62	1200	NC 002134.1
	TnpA21-R	TCA GCC CGG CAT GCA CGC G			
*tnpA* of Tn*7*	TnA7-F	CCCAGCAATAAAAGAGCTCATTGAGCAAGC	55	738	FJ914220.1
	TnA7-R	TATCTAGAAACAGAGTGTCTTG			
**(fluoro)quinolone resistance genes**					
*qnrA*	qnrA-F	TTCAGCAAGAGGATTTCTCA	55	627	AY070235
	qnrA-R	GGCAGCACTATTACTCCCAA			
*qnrB*	qnrB-F	CCTGAGCGGCACTGAATTTAT	60	408	DQ351241
	qnrB-R	GTTTGCTGCTCGCCAGTCGA			
*qnrS*	qnrS-F	CAATCATACATATCGGCACC	60	641	AB187515
	qnrS-R	TCAGGATAAACAACAATACCC			
*aac(6′)-Ib-cr*	aac(6′)-Ib-cr-F	TTGCGATGCTCTATGAGTGGCTA	55	482	AAL93141.1
	aac(6′)-Ib-cr-R	CTCGAATGCCTGGCGTGTTT			
	aac(6′)-Ib-cr (sequencing)	CGTCACTCCATACATTGCAA			
***bla*genes**					
*blaTEM*	TEM-F	ATGAGTATTCAACAT TTC CG	55	840	EF125012
	TEM-R	CCAATGCTTAATCAG TGA GG			
*blaSHV*	SHV-F	TTCGCCTGTGTATTATCTCCCTG	50	854	AF148850
	SHV-R	TTAGCGTTGCCAGTGYTCG			
*blaCTX-M*	CTX-M-F	ATGTGCAGYACCAGTAARGTKATGGC	60	593	Y10278
	CTX-m-R	TGGGTRAARTARGTSACCAGAAYCAGCGG			
*blaCMY*	CMY-F	ATGATGAAAAAATCGTTATGC	55	1200	U77414
	CMY-R	TTGCAGCTTTTCAAGAATGCGC			
*blaOXA-1*	OXA-1 F	ATGAAAAACACAATACATATCAACTTCGC	62	820	JO2967
	OXA-1R	GTGTGTTTAGAATGGTGATCGCATT			
*blaOXA-2*	OXA-2 F	ACGATAGTTGTGGCAGACGAAC	62	602	AF300985
	OXA-2R	ATYCTGTTTGGCGTATCRATATTC			

Primers used for screening various genetic elements and for interrogating physical linkages between different genetic elements and between such elements and *bla* genes or (fluoro)quinolone resistance genes.

Y = T or C, R = G or A, S = G or C, K = G or T.

### Detection of *aac(*6’*)-lb-cr* and *qnr* genes

Screening for *aac(*6*′)-Ib-cr* gene that confers cross-resistance to fluoroquinolones and aminoglycoside was done using a combination of PCR, RFLP and sequencing as described by Park *et al.*[41]. The isolates were also screened for genes conferring resistance to quinolones: - *qnr*A, *qnr*B and *qnr*S using PCR and sequencing strategies previously described by Wu *et al.*[42].

### Interrogation for physical linkages between genetic elements and resistance genes

Physical linkages between integron and the transposons were determined using a combination of published primers targeting 5’-conserved sequences (5’-CS) of class 1 integrons and those targeting the *tnpM* of Tn*2* or those specific for *tnpA7* of Tn*7*, Figure 1*.* A combination of primers targeting IS elements and those targeting the 5’-CS or the 3’-termini of integrons were used for interrogation for physical linkages between integrons and IS elements. A combination of primers specific for various genetic elements and consensus primers for *bla*_*SHV*_ or *bla*_*TEM*,_[43,44], *bla*_*CTX-M*_[45], *bla*_*CMY*_[46] and *bla*_*OXA*_[47,48] were used for determination of physical linkages between *bla* genes and different genetic elements. Primers for *aac(*6’*)-lb-cr* and *qnr* genes were used in combination with those for different genetic elements to analyze for their physical association. A long-range polymerase [LongAmp® Taq DNA Polymerase, (New England Biolabs, USA)] was used in all reactions for physical linkages. A slow ramping rate of between 0.2°C/sec and 0.3°C/sec was set for the annealing step. The extension time was set at 72°C for 2 min and a final extension of 72°C for 15 min was carried out after 35–40 cycles of denaturation, annealing and extension.

### Conjugation experiments

Conjugation experiments using sodium azide resistant *E. coli* strain *J*53 as the recipient were done as previous described [49]. Susceptibility to antimicrobials and determination of genetic element content of the transconjugants was determined using similar methods as those used for the corresponding donor strains. Plasmid incompatibility groupings were determined using the scheme of Carattoli *et al.*[50].

### Statistical analysis

For the purpose of analysis, both intermediate and resistant results for antibiotic susceptibility testing were grouped together as “resistant”. Differences in proportion of isolates bearing different elements was analyzed using the Chi test (*χ*2) while the Fisher’s exact test was used for smaller sample sizes. The Odds Rations (OR) and the 95% confidence intervals (CIs) accompanying the *χ*2 tests were determined using the approximation of Woolf. The null hypothesis was rejected for values of p ≥ 0.05. Statistical analysis was performed using Statgraphics plus Version 5 (StatPoint Technologies, INC, Warrenton, VA, USA).

## Competing interests

None of the authors have competing interests.

## Authors’ contributions

JK designed the study, carried out the experiments and wrote the manuscript. SK, BM and PB participated in manuscript write-up and review. All authors read and approved the final manuscript.

## Authors’ information

JK and SK are research scientists at the Kenya Medical Research Institute (KEMRI). BMG is Professor at the K.U.Leuven (Faculty of Bioscience Engineering) while PB is a Senior Research Scientist at the Veterinary and Agrochemical Research Centre (VAR).
